# Using Flexible-Printed Piezoelectric Sensor Arrays to Measure Plantar Pressure during Walking for Sarcopenia Screening

**DOI:** 10.3390/s24165189

**Published:** 2024-08-11

**Authors:** Shulang Han, Qing Xiao, Ying Liang, Yu Chen, Fei Yan, Hui Chen, Jirong Yue, Xiaobao Tian, Yan Xiong

**Affiliations:** 1 College of Mechanical Engineering, Sichuan University, Chengdu 610065, China; hsl@stu.scu.edu.cn; 2 College of Mechanical and Electrical Engineering, Chengdu University of Technology, Chengdu 610059, China; m_xiao@stu.cdut.edu.cn; 3 College of Architecture and Environment, Sichuan University, Chengdu 610065, China; liangying@scu.edu.cn (Y.L.); yu_chen@scu.edu.cn (Y.C.); 4 Chongqing Municipality Clinical Research Center for Geriatric Diseases, Chongqing University Three Gorges Hospital, School of Medicine, Chongqing University, Chongqing 404000, China; fei.yan@cqu.edu.cn; 5 Department of Senile Medical, The Affiliated Traditional Chinese Medicine Hospital of Southwest Medical University, Luzhou 646000, China; huige@swmu.edu.cn; 6 Department of Geriatrics, West China Hospital, Sichuan University, Chengdu 610041, China

**Keywords:** piezoelectric sensor, flexible printing, dynamic plantar pressure monitoring, sarcopenia screening, machine learning

## Abstract

Sarcopenia is an age-related syndrome characterized by the loss of skeletal muscle mass and function. Community screening, commonly used in early diagnosis, usually lacks features such as real-time monitoring, low cost, and convenience. This study introduces a promising approach to sarcopenia screening by dynamic plantar pressure monitoring. We propose a wearable flexible-printed piezoelectric sensing array incorporating barium titanate thin films. Utilizing a flexible printer, we fabricate the array with enhanced compressive strength and measurement range. Signal conversion circuits convert charge signals of the sensors into voltage signals, which are transmitted to a mobile phone via Bluetooth after processing. Through cyclic loading, we obtain the average voltage sensitivity (4.844 mV/kPa) of the sensing array. During a 6 m walk, the dynamic plantar pressure features of 51 recruited participants are extracted, including peak pressures for both sarcopenic and control participants before and after weight calibration. Statistical analysis discerns feature significance between groups, and five machine learning models are employed to screen for sarcopenia with the collected features. The results show that the features of dynamic plantar pressure have great potential in early screening of sarcopenia, and the Support Vector Machine model after feature selection achieves a high accuracy of 93.65%. By combining wearable sensors with machine learning techniques, this study aims to provide more convenient and effective sarcopenia screening methods for the elderly.

## 1. Introduction

Sarcopenia, characterized by the progressive loss of skeletal muscle mass, strength, and function, is a prevalent age-related syndrome associated with increased morbidity of other diseases and mortality among older adults [1,2,3]. The consequences of sarcopenia extend beyond mere physical impairment, impacting overall health, independence, and quality of life [4]. Accompanied by the trend of global aging, the burden of sarcopenia is expected to rise, highlighting the urgent need for effective screening and intervention strategies to mitigate its adverse outcomes [5]. Early screening of sarcopenia is critical for timely intervention and improved clinical outcomes. However, traditional methods for assessing muscle mass and function, such as dual-energy X-ray absorptiometry (DXA), bioelectrical impedance analysis (BIA), computed tomography (CT), and grip strength measurements, are often impractical for large-scale screening and long-term monitoring due to their cost, complexity, and requirement for specialized equipment and expertise [6,7,8,9]. Consequently, there is an increasing need for accessible, cost-effective, and convenient screening tools that can accurately identify individuals at risk of sarcopenia in community surroundings.

Plantar pressure, as a kind of biomechanical parameter, has gradually emerged as a potential indicator for sarcopenia screening due to its close association with musculoskeletal function, gait performance, and overall balance [10,11,12]. Plantar pressure appears to be mostly related to an individual’s balance ability and may be more effective in evaluating the imbalanced distribution of plantar pressure. Therefore, it can be used to determine deformities or abnormalities in foot structure [13,14]. Dynamic plantar pressure refers to the pressure exerted by an individual during walking or running, which changes more dramatically during exercise and may be more effective in monitoring gait abnormalities and sports injuries [15]. During a gait cycle, the foot acts as a dynamic platform for weight-bearing and propulsion, with the plantar surface experiencing varying levels of pressure distribution corresponding to different phases of movement [16]. Alterations in plantar pressure patterns, including abnormal peak pressures or pressure–time integral values, are possibly indicative of gait abnormalities and compromised musculoskeletal function [17,18,19].

Commonly used methods for measuring plantar pressure, such as pressure-sensitive pads or pressure plates, are restricted in their ability to capture dynamic pressure changes during walking due to their limited size and are generally not suitable for large-scale screening and long-term monitoring. Furthermore, these methods often require controlled laboratory conditions and trained personnel for data acquisition and analysis, making them impractical for use outside of clinical or research settings [20,21]. At present, the device suitable for real-time monitoring and data transmission is the wearable intelligent insole system, which usually integrates sensors and wireless transmission modules with advantages such as portability, pressure resistance, and reliability [22,23]. In recent years, there has been growing interest in the development of wearable sensor technologies for assessing plantar pressure during walking. These technologies offer several advantages over traditional methods, including portability, ease of use, and the ability to capture real-time data in diverse environments [24]. Among these technologies, flexible sensors have emerged as a promising approach for pressure measurement due to their light weight, conformal property, and ability to capture dynamic pressure changes with high sensitivity and spatial resolution, which can provide an efficient and integrated manufacturing method for intelligent pressure insoles [25,26,27,28,29].

A piezoelectric sensor, as a type of sensor device that converts pressure responses into charge signals, has advantages such as fast response speed, wide frequency response range, and high linearity, and is therefore considered an excellent device for measuring dynamic pressure changes [30,31]. A flexible-printed piezoelectric sensor array is a promising approach for manufacturing intelligent insole systems. It consists of thin-film piezoelectric materials integrated onto flexible substrates, allowing them to conform to the contours of the foot and accurately measure pressure distribution during walking. These sensor arrays can be seamlessly integrated into footwear or insole systems, enabling unobtrusive and continuous monitoring of plantar pressure during daily activities. Moreover, the combination of sensor arrays with wireless connectivity and real-time data transmission facilitates remote monitoring and timely intervention by healthcare providers, enhancing the accessibility and effectiveness of sarcopenia screening.

The main purpose of this article is to explore the potential of dynamic plantar pressure in effective and convenient screening for sarcopenia. Therefore, in this article, we propose a wearable flexible-printed piezoelectric sensing array based on barium titanate thin films for dynamic plantar pressure monitoring during walking. We used a flexible printer for flexible substrate packaging and the dispensing printing of flexible electrode arrays, integrating the barium titanate film array into the flexible substrates, which could greatly improve the compressive strength and maximum measurement range of the sensing array. The charge signal of the sensor was converted into a voltage signal input into the microprocessor ESP32 through a signal conversion circuit, which was then transmitted to a mobile phone via Bluetooth. We obtained the average voltage sensitivity (4.844 mV/kPa) of the entire pressure sensing array through cyclic loading after signal processing and transmission using linear fitting. At the same time, we extracted some dynamic plantar pressure features, including peak pressures, from patients with sarcopenia and control participants during a 6 m walk, as well as these features after weight calibration. In order to explore the potential of these features in screening for sarcopenia, we first explored the significance of these features between the sarcopenia group and the control group through statistical analysis, and then used machine learning algorithms to classify and screen sarcopenia cases using the selected features, achieving the highest accuracy (93.65%) with SVM. By combining wearable sensor technologies with machine learning methods, we aim to contribute to the development of more convenient and effective screening and management methods for sarcopenia in the elderly.

## 2. Materials and Methods

### 2.1. Piezoelectric Film

Piezoelectric thin films are considered good force–electricity converters based on their inherent piezoelectric effect. The piezoelectric effect is a phenomenon that refers to the polarization of piezoelectric materials under external pressures, ultimately resulting in the generation of positive and negative charges on the top and bottom surfaces. Collecting or analyzing the charges generated by external pressures reveals the potential of piezoelectric thin films as effective energy generators or pressure sensors. Barium titanate is a lead-free inorganic piezoelectric ceramic material with a high piezoelectric coefficient. However, general barium titanate piezoelectric ceramics are relatively fragile and have poor mechanical properties. At the same time, by electrospinning, barium titanate can be made into fibrous thin films, which can improve its mechanical properties to a certain extent. For barium titanate thin films, they generally work in the direction of the piezoelectric coefficient d_33_. Under positive pressure in this direction, the charge output of barium titanate thin films is defined as q = d_33_X, where q is the charge density and X is the normal stress.

### 2.2. Fabrication of Plantar Pressure System with Sensor Arrays

**Figure 1:**
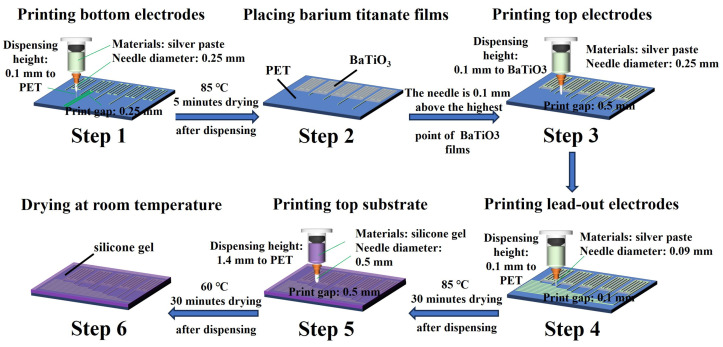
Schematics of the fabrication process of the plantar pressure system with sensor arrays.

**Figure 2:**
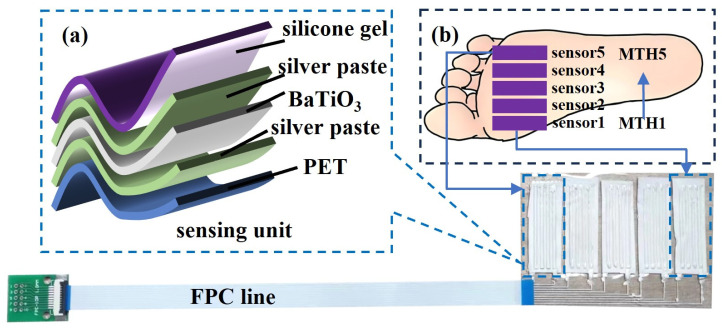
Schematic diagram of the plantar pressure arrays: (**a**) the specific structure of each individual sensing unit; (**b**) the placement position of the sensor on the sole of the foot.

During dynamic plantar pressure measurement, localized pressures on the plantar surface may exceed 100 kPa. However, directly encapsulating piezoelectric thin film materials on the plantar surface may lead to material damage and failure during the measurement process. Flexible printing technology is a manufacturing process that utilizes flexible materials and high-precision printing techniques to directly print electronic components, sensors, and other functional parts onto flexible substrates. The benefit of flexible packaging for fragile materials lies in its capacity to directly print electronic components onto soft, stretchable substrates, thereby enhancing their flexibility and adaptability to complex surfaces and irregular shapes. Moreover, it mitigates the risk of damage to fragile materials during the assembly process. This study employs a flexible printer (MP1100, Prtronic in Shanghai, China) to manufacture and encapsulate a pressure array for the sole of the foot, ensuring its stability under significant pressure. The entire manufacturing process of the plantar pressure array is illustrated in Figure 1. Initially, conductive silver paste (Base-CD01, Prtronic in Shanghai, China) is dispensed onto a PET substrate using a printing tube with a needle diameter of 0.25 mm at a height of 0.1 mm above the PET substrate and with a print gap set to 0.25 mm. Subsequently, the electrode array is heated and dried at 85 °C for 5 min to achieve partial drying. Following this, barium titanate films with a size of about 1.4 × 4 × 0.17 cm are placed on each bottom electrode to ensure close contact. The barium titanate thin film used in this article was obtained through electrospinning based on our previous work [32]. The needle is then raised to 0.1 mm above the highest point of the uneven surface of the BaTiO_3_ film, and the same process is repeated for printing the top electrode array, with a print gap adjusted to 0.5 mm. Due to the diffusion effect of silver paste, the actual thickness of the silver paste electrodes will be less than their corresponding set heights. The thickness of the bottom silver paste electrode is about 80 μm, and the thickness of the top silver paste electrode is about 281 μm (measured at a relatively flat position). Next, a needle with a diameter of 0.09 mm is used to print silver paste electrodes for lead-out lines at a height of 0.1 mm and with a print gap maintained at 1 mm to match the electrode spacing of the external FPC (Flexible Printed Circuit) line for connection. Subsequently, all silver paste electrodes are heated at 85 °C for 30 min to ensure complete drying and the tight connection of all contact parts. Finally, the top layer of silicone (Test-D1, Prtronic in Shanghai, China) is encapsulated at a height of 1.4 mm from the PET substrate using a printing tube with a needle diameter of 0.51 mm and with a print gap set to 0.51 mm, ensuring that the total height of the plantar pressure array is approximately 1.5 mm. Then, the printed sensing array is heated at 60 °C for 30 min and dried at room temperature (25 °C) for 4 h to obtain the plantar pressure measurement array shown in Figure 2a. The specific structure of each individual sensing unit with a sandwich design obtained by flexible printing is depicted in Figure 2b: a bottom layer of PET (polyethylene terephthalate) film, with a barium titanate piezoelectric film sandwiched between silver paste electrodes on both surfaces, all encapsulated in a layer of silicone gel.

**Figure 3:**
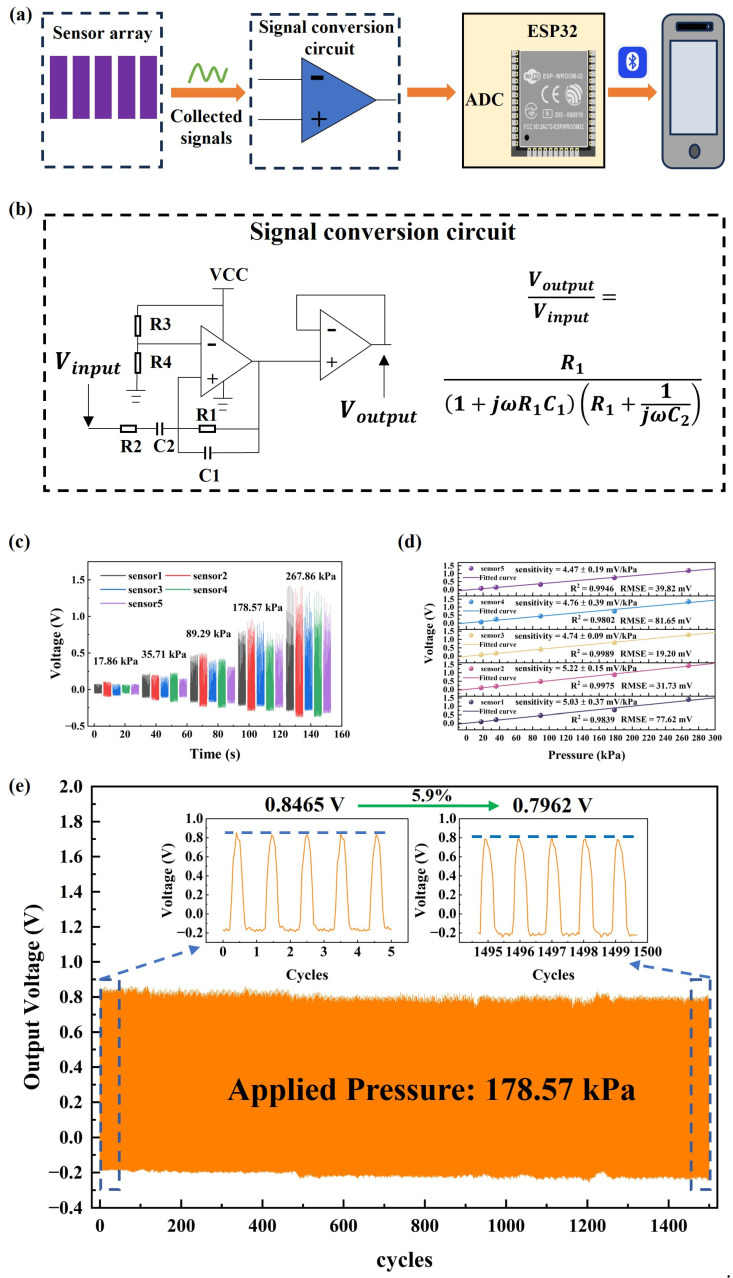
(**a**) Schematic diagram of the entire plantar pressure measurement system: (**b**) the signal processing circuit of a single sensing unit; (**c**) the voltage output of the sensing array under different normal pressures; (**d**) separate voltage sensitivity results of the 5 sensing units; (**e**) the output stability of the sensing array under a pressure of 178.57 kPa.

The size of the entire plantar pressure array is 8 × 6 × 0.15 cm, and it is fixed on the forefoot of the left foot insole since the peak of dynamic plantar pressure during walking usually occurs at the forefoot region [33]. It is used as a proof of concept to demonstrate the functioning capability of the proposed device and construct a promising approach for sarcopenia screening by monitoring plantar pressure in the forefoot during walking. While walking, the pressure on the five metatarsal bones (MTH1–MTH5) of the left forefoot varies significantly. This dynamic plantar pressure can indicate various pathological conditions, such as deformed feet, obesity, and diabetes. The plantar pressure sensing array developed in this study analyzes the dynamic pressure in these five areas and extracts relevant features for sarcopenia screening using machine learning methods. The positions of the sensor units relative to the five metatarsal bones are shown in Figure 2c. Figure 3a illustrates a schematic of the entire plantar pressure measurement system, which transmits the pressure signals generated during walking to an ESP32 (ESP32-WROOM-32E, ESPRESSIF in Shanghai, China) microcontroller via a signal processing circuit and then sends the data to a mobile phone via Bluetooth for further analysis and processing. Figure 3b depicts the signal processing circuit of a single sensing unit, which mainly comprises a charge amplification circuit and a voltage follower. The amplitude–frequency characteristic equation of the input and output of the charge amplifier is also described in Figure 3b. The amplification chips applied in the charge amplification modules are all TL072 (Sekorm Advanced Technologies in Shenzhen, China), which is a chip with low noise, high input impedance, and low bias current. Meanwhile, there is also a power module with a lithium battery to supply TL072 and ESP32. The actual photography of the entire prototype system is shown in Figure S1a. Sensitivity calibration of the overall plantar pressure measurement system is performed by using an exciter (HEV-20, Foneng in Nanjing, China) that generates cyclic positive pressure with the effective area of 4×1.4 cm^2^. The exciter is powered by a power amplifier (HEAS-20, Foneng in Nanjing, China). Specifically, the exciter generates cyclic positive pressures that act on each sensing unit. A commercial piezoelectric sensor (3A105, Donghua in Taizhou, China) is used to measure the pressure exerted during this process, and the corresponding voltage output of this system during the cyclic process is obtained. A photo of the experimental test bench is shown as Figure S1b in the Supplementary Materials. The voltage output of the sensing array under different normal pressures is shown in Figure 3c. Linear fitting is performed between the collected output voltage and pressure to obtain separate voltage sensitivity results, as shown in Figure 3d, and the average voltage sensitivity is 4.844 mV/kPa. Figure 3e shows the output stability of the sensing array under the pressure of 178.57 kPa, with only a 5.9% decrease in the output voltage compared to the original. The response and recovery time are approximately 35 ms and 104 ms, as shown in Figure S2a. From Figure S2b, we can see that the minimum detection limit of the sensor is 1.5 kPa. Figure S3 shows the bending characteristic of the sensor, where the output voltage is increasing by the increase in the bending angles.

### 2.3. Data Collection of Plantar Pressure

**Figure 4:**
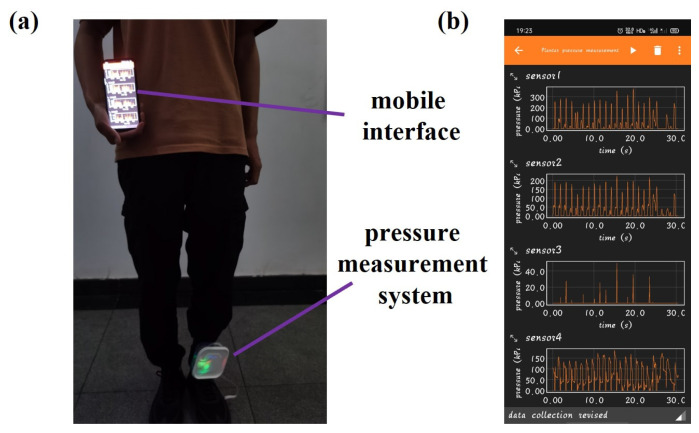
(**a**) Schematic diagram of plantar pressure measurement process. (**b**) The plantar pressure curve graphs received through Bluetooth during the measurement process.

The plantar pressure measurement array was integrated into the insole of sports shoes to measure the dynamic pressure of control participants and sarcopenia patients during walking. This study recruited 51 participants aged above 55 to participate in dynamic plantar pressure measurements, including 32 control participants and 19 sarcopenia patients. Due to the width of the plantar pressure array used for measurement being approximately 8 cm, only participants with foot sizes within the range of 38–43 yards (European standard) were recruited. The exclusion criteria for plantar pressure testing are as follows: (1) individuals with other diseases (such as knee osteoarthritis) that affect walking ability, (2) individuals with cognitive impairment or inability to cooperate, and (3) individuals with foot deformities. This study was approved by the Ethics Committee of Sichuan University (Ethics Approval Number: 2021[96]), and informed consent was obtained from all participants. The entire testing process was carried out in accordance with the principles expressed in the Helsinki Declaration. Table 1 shows the basic characteristics of the participants. During the testing process, participants were required to wear shoes integrated with the constructed pressure measurement system with the plantar pressure array placed only on the left foot, as shown in Figure 4a, and they were asked to walk 6 m at a normal speed in a designated area. The sampling frequency of the pressure signal during the measurement process was 100 Hz, while all collected data were transmitted through Bluetooth and the original dynamic curve was displayed on the mobile phone. The plantar pressure curve graphs received through Bluetooth during the measurement process are shown in Figure 4b. The diagnostic methods for sarcopenia were defined according to the guidelines of “Asian working group for Sarcopenia: 2019 consensus update on Sarcopenia diagnosis and treatment” (AWGS2019) [34]. The final results were determined based on the diagnostic criteria in AWGS2019.

### 2.4. Data Processing

After collecting pressure signals from the five metatarsals of each participant, the signals were first filtered using a 10 Hz low-pass filter to remove noise interference. Additionally, due to the varying weights of the participants, we also obtained plantar pressure features with weight calibration by dividing the measured pressure value by the participant’s weight. By comparing the effectiveness of these two types of pressure data in screening for sarcopenia, we aimed to determine whether weight calibration has a significant effect on the application of dynamic plantar pressure in medical diagnosis. The category label data were converted into numerical data for subsequent machine learning classification. In the diagnostic results, control participants were labeled as 0, while sarcopenia patients were labeled as 1. Depending on the distribution of all collected feature data, either the Student’s *t*-test or the Mann–Whitney U-test was used to statistically analyze the differences between control participants and sarcopenia patients. A statistical analysis was conducted using SPSS (IBM SPSS Statistics 27.0.1). If *p* < 0.05, it is considered that there is a statistical difference between the two groups of data.

### 2.5. Feature Extraction and Selection

**Figure 5:**
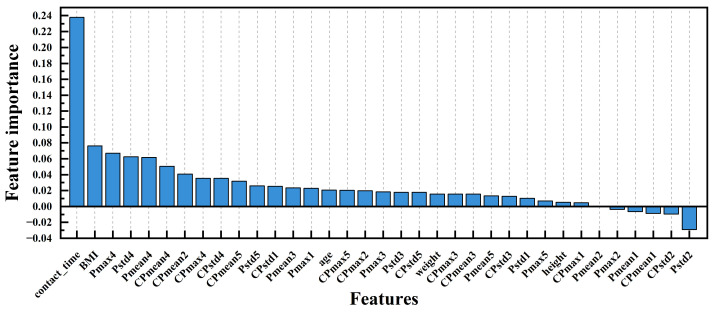
The importance ranking of the 35 features calculated by the ReliefF algorithm.

The dynamic plantar pressure curve of participants during a 6 m walk can be collected through a plantar pressure array. Based on the dynamic pressure curve, we can extract the average peak pressure (Pmax), average pressure (Pmean), pressure standard deviation (Pstd), contact time (the contact time with ground of the left forefoot) of the entire process, as well as the calibrated peak pressure (CPmax), calibrated average pressure (CPmean), and calibrated pressure standard deviation (CPstd) after weight calibration. The calculation of plantar pressure features here is entirely based on all pressure samples with values > 0. There are a total of 31 features corresponding to the measurement areas of the 5 sensors in the forefoot, as follows: Pmax1–Pmax5, Pmean1–Pmean5, Pstd1–Pstd5, CPmax1–CPmax5, CPmean1–CPmean5, CPstd1–CPstd5, and contact time. The four physiological basic features that may be related to sarcopenia (height, weight, BMI, and age) were also included in the extracted features, resulting in a total of 35 extracted features. The ReliefF algorithm is used for feature selection [35], and under this algorithm, the top 7, 14, 21, 28, and 35 features based on feature importance are selected for model training and validation, in order to obtain a model with the best classification performance after feature selection. The importance ranking of the 35 features calculated by the ReliefF algorithm is shown in Figure 5.

### 2.6. Machine Learning Models for Sarcopenia Screening

This study employed 5 types of machine learning models, including Decision Tree (DT), Logistic Regression (LR), Naive Bayes (NB), Support Vector Machine (SVM), and K-Nearest Neighbors (KNN). Note that there is a slight imbalance in the distribution of control participants and sarcopenia patients in the original data. Here, an oversampling method is adopted to solve the imbalance problem and achieve better model performance. Synthetic Minority Oversampling (SMOTE), as a commonly used oversampling method, is used to increase the size of minority class data to solve the problem of imbalanced data in this study without changing the distribution in the original data [36]. It synthesizes new samples between two minority class samples through linear interpolation instead of simply creating samples, effectively alleviating the overfitting problem caused by random oversampling. In Python 3.11.2, 10-fold cross validation is used to train and evaluate these models, which can also reduce model overfitting and enhance generalization ability.

The performance of machine learning classification models is evaluated using six different metrics: accuracy (ACC), precision (PRE), recall (REC), specificity (SPE), F1 score (F1), and the area under the receiver operating characteristic curve (AUC) [37]. Accuracy (ACC) refers to the proportion of correctly predicted samples in a model to the total number of samples and is one of the most commonly used indicators to evaluate machine learning performance. However, when the overall sample categories are extremely imbalanced, ACC can easily lead to a misjudgment of model performance. In cases where the sample categories are relatively balanced, ACC can be an effective evaluation indicator. Precision (PRE) refers to the proportion of samples predicted as positive by the model to those that are truly positive, representing the model’s accuracy in classifying positive examples. Recall (REC) represents the proportion of true positive cases to all actual positive cases in the predicted results. Many practical application scenarios require detecting all categories that need to be predicted as much as possible, such as earthquake prediction. Specificity (SPE) indicates the proportion of samples that are correctly predicted as negative examples among the true negative examples. The F1 score (F1) is the harmonic mean of precision and recall, used to comprehensively evaluate the performance of the model. Meanwhile, the area under the receiver operating characteristic curve (AUC) represents the area under the ROC curve, reflecting the model’s generalization ability. Comprehensively considering these commonly used indicators in machine learning performance evaluation can help us choose the model with the best performance. The metrics selected for performance evaluation of classification models are defined as follows:

(1) $$\begin{array}{c} ACC=\frac{TP+TN}{TP+TN+FP+FN} \end{array}$$

(2) $$\begin{array}{c} PRE=\frac{TP}{TP+FP} \end{array}$$

(3) $$\begin{array}{c} REC=\frac{TP}{TP+FN} \end{array}$$

(4) $$\begin{array}{c} SPE=\frac{TN}{TN+FP} \end{array}$$

(5) $$\begin{array}{c} F1=\frac{2\cdot Precision\cdot Recall}{Recall+Precision}=\frac{2TP}{2TP+FP+FN} \end{array}$$

TP: The real label is a positive example, and the predicted label is a positive example. FP: The real label is negative, and the predicted label is positive. TN: The real label is negative, and the predicted label is negative. FN: The real label is a positive example, and the predicted label is a negative example.

## 3. Results

**Figure 6:**
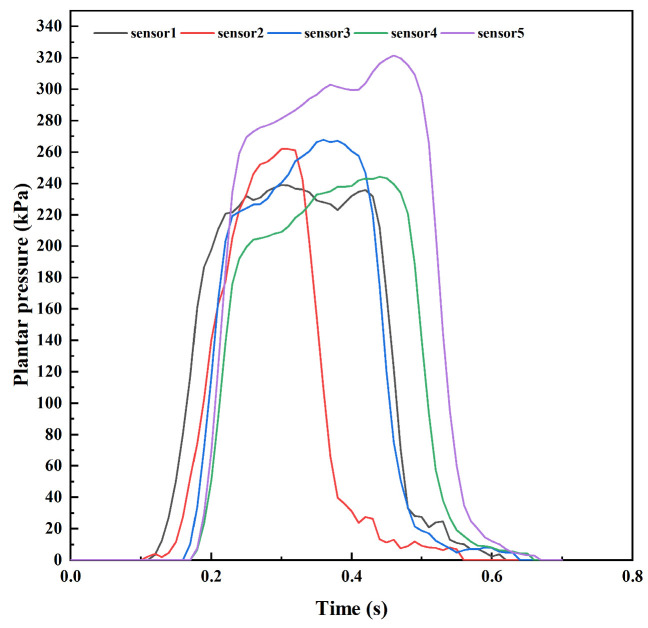
An example of dynamic plantar pressure curves measured by piezoelectric sensor arrays at five metatarsal bones during a gait cycle.

**Figure 7:**
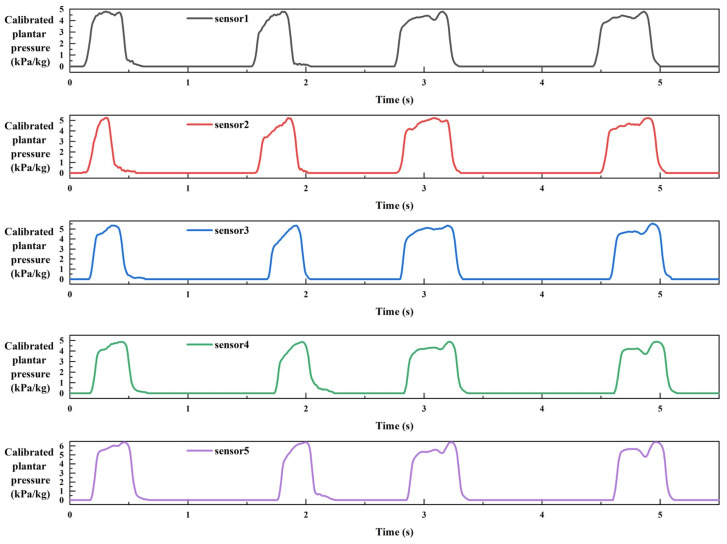
The dynamic plantar pressure curves of a participant after weight calibration throughout the entire walking period.

Based on the calibrated average voltage sensitivity, the measured voltage signal can be converted into dynamic plantar pressure signals during walking. Figure 6 shows an example of dynamic plantar pressure curves measured by piezoelectric sensor arrays at five metatarsal heads during a gait cycle. It can be seen that the moment when these five areas reach peak pressure during walking is not the same, and there is a short time interval. The dynamic plantar pressure curves of a participant after weight calibration throughout the entire walking period are illustrated in Figure 7. Due to the thickness of the pressure array manufactured in this study, the soles of the feet of the participants are tightly attached to the pressure array during walking. Therefore, during the stable walking process of the subjects, the trends of pressure changes in the five areas of the forefoot are a little similar, resulting in similar dynamic change curves by the five sensors in Figure 7. However, due to the physiological structure of the human body and the individual’s walking habits, the peak pressures on these five areas during walking are generally not the same.

Table 2 reports the statistical analysis results of the extracted features including the dynamic plantar pressure features collected throughout the testing process, with all data stratified by control participants and sarcopenia patients. The features in Table 2 were first tested for normality. The data that conform to the normal distribution are represented as mean ± standard deviation, while the data that do not conform to the normal distribution are represented as median (interquartile range). As shown in Table 2, for the four physiological characteristics collected, three of them (age, weight, and BMI) showed significant differences between the sarcopenia group and the control group, while height did not. For the 15 features before weight calibration, 9 features (Pmax1–Pmax3, Pmean1–Pmean2, Pmean5, Pstd1, and Pstd3–Pstd4) showed significant differences between the control group and the sarcopenia group, while other features showed no significant differences. After weight calibration, there were significant differences in the 11 features (CPmax1–CPmax2, CPmax5, CPmean1–CPmean5, CPstd1–CPstd2, and CPstd5) between the control group and the sarcopenia group. Before weight calibration, the sarcopenia group also showed higher peak pressures at the position of MTH1 and MTH2 but lower peak pressures at the position of MTH3 compared to the control group (*p* < 0.05). After weight calibration, the sarcopenia group showed higher calibrated peak pressures at the position of MTH1, MTH2, and MTH5 compared to the control group (*p* < 0.05). Meanwhile, there was a significant difference in the contact time between the sarcopenia group and the control group.

**Figure 8:**
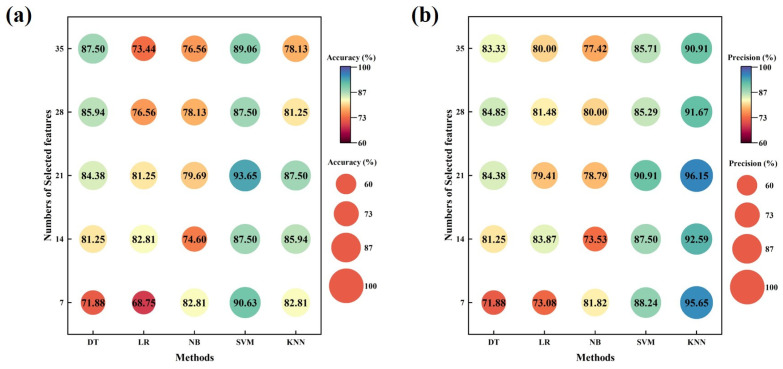

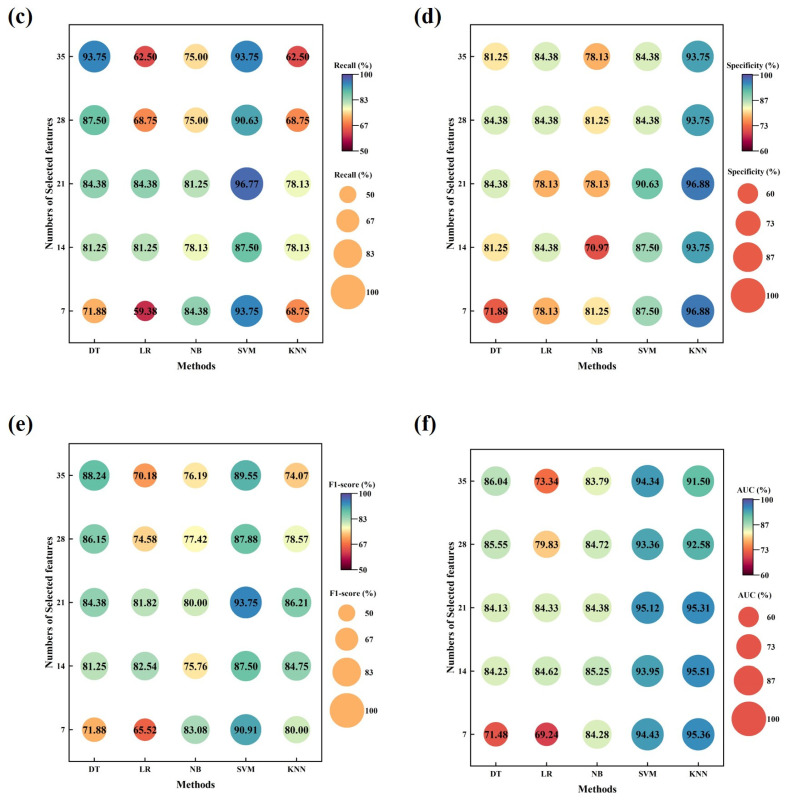
The performance evaluation of 5 machine learning models using different numbers of the selected features: (**a**) accuracy; (**b**) precision; (**c**) recall; (**d**) specificity; (**e**) F1 score; (**f**) AUC.

Figure 8a–f illustrate the performance evaluation of the five machine learning models using the selected top features with the quantity of 7, 14, 21, 28, and 35, respectively. The meaning of the *y*-axis of Figure 8a–f refers to the number of features we selected for machine learning training, and the values inside the circles in Figure 8 represent the various evaluation indicators for the machine learning models, which are used to compare the comprehensive performance of different machine learning models with different numbers of features and select the best model for screening for sarcopenia. Without feature selection, SVM performed the best in accuracy (89.06%), recall (93.75%), F1 (89.55%), and AUC (94.34%) by evaluating with all the evaluation indicators (ACC, PRE, REC, SPE, F1, and AUC), while the precision and specificity were all a little lower than the KNN model. On the contrary, when all features were used, LR exhibited the lowest performance with the lowest accuracy (73.44%), recall (62.50%), F1 (70.18%), and AUC (73.34%). After using feature selection algorithms, DT, LR, NB, SVM, and KNN achieved their best performance when the number of selected features was 35, 14, 7, 21, and 21, respectively. Among them, SVM with 21 selected top features showed the best performance among all models, with high accuracy (93.65%), high recall (96.77%), and a high F1 value (93.75%).

## 4. Discussion

The purpose of this article is to measure the dynamic plantar pressure values of control individuals and sarcopenia patients during walking, extract basic features about plantar pressure through dynamic pressure curves, and ultimately use these features to construct machine learning models for effective screening of sarcopenia. As age increases gradually, the muscle mass and muscle function of patients with sarcopenia will gradually decrease, which may lead to changes in foot structure and motor function. Patients with sarcopenia may experience slow walking speed, reduced walking balance, and decreased physical strength [38]. As shown in Table 2, the age, weight, and BMI of the sarcopenia group were significantly lower than those of the control group, which is consistent with the conclusions of the reference literature [39]. Additionally, the peak pressures of sarcopenia patients in the MTH1, MTH2 zone are higher than that of control individuals, while the peak pressures at MTH3 are lower than that of control individuals. This may attribute to the decrease in walking speed in patients with sarcopenia, which leads to a decrease in plantar pressure of some areas [40]. Meanwhile, the difficulty in maintaining balance during walking results in an increase in plantar pressure of the other areas. The increases in plantar pressure also put urgent requirements on the shoes worn in daily life, and the study of peak plantar pressure during walking can provide effective guidance for rehabilitation footwear products [41]. The decrease in walking speed may lead to longer contact time during walking for individuals with sarcopenia [42], as shown in Table 2. According to Table 2, some features after weight calibration showed significant differences between control individuals and sarcopenia patients, while there were no differences before calibration. This indicates that in some medical applications, some features of plantar pressures cannot show significant differences in disease diagnosis due to weight differences between individuals. The plantar pressure after weight calibration may play a more significant role in some medical applications. As shown in Figure 8 in this study, machine learning models for screening for sarcopenia were established using the extracted features, and high accuracies were achieved. However, in the actual process, due to the different walking habits and physiological structures of participants, some of their feature data do not fully conform to the constructed models, possibly resulting in low recall or specificity values. Therefore, due to the complex structure of the human body and its complex biomechanical system, there is still a long way to go in discovering and studying features or biomechanical indicators that have a prominent recognition effect between the sarcopenia group and the control group.

Table S1 shows the required components and approximate cost for creating a prototype for measuring plantar pressure established in this article. The total cost of this prototype system is around USD 35 and only includes eight circuit components, greatly reducing the design complexity. Meanwhile, after setting up the microcontroller, one must simply turn on the power supply and Bluetooth of the phone to turn on the system for real-time dynamic plantar pressure monitoring, making it easy to operate. Table 3 compares the plantar pressure prototype system proposed in this article for screening for sarcopenia with some existing plantar pressure measurement devices used for sarcopenia screening or other medical applications, as well as commercial devices commonly used for sarcopenia screening. The comparative content presented presents the advantages and disadvantages of each method, mainly evaluating aspects such as preparation methods, sensitivity, response time, cost, portability, complexity, wireless transmission, and real-time monitoring. The comparison shows that the plantar pressure measurement system proposed in this article is a low-cost, fast response, and convenient prototype system for screening for sarcopenia, which can provide real-time and efficient services for sarcopenia patients.

By combining dynamic plantar pressure measurement using plantar pressure arrays with machine learning classification methods, monitoring and early screening for sarcopenia can be achieved. However, there are several limitations of this study that need to be addressed in future research. Firstly, the machine learning screening model used in this study should be validated in a wider population to pursue better generalization ability [43]. At the same time, patients with sarcopenia and control individuals in different regions may have different characteristics of plantar pressure [44], so the effective features based on dynamic plantar pressure monitoring for early screening of sarcopenia may vary in different regions. Additionally, the pressure areas of the sole collected in this study are limited, and more valuable features may be obtained by studying the pressure distribution of the entire sole, but this will inevitably lead to the complexity of data analysis and classification models. Finally, this study only involved early screening for sarcopenia, but did not involve the classification of the severity of sarcopenia.

## 5. Conclusions

This study demonstrates the effectiveness of using dynamic plantar pressure for early screening of sarcopenia. The use of a flexible-printed piezoelectric sensor array to measure dynamic plantar pressure, combined with machine learning classification models, enables real-time monitoring and screening of sarcopenia patients. By fabricating the sensing arrays with barium titanate thin film and employing signal conversion circuits, we achieved efficient data acquisition and transmission to mobile devices. This integrated approach of wearable sensor technology and machine learning holds considerable potential for improving sarcopenia screening and management strategies in elderly populations. Further research and validation studies are required to establish the clinical utility and effectiveness of this approach in larger groups.

## Figures and Tables

**Table 1:** Basic characteristics of the participants.

Characteristics	Non-Sarcopenia (Numbers = 32)	Sarcopenia (Numbers = 19)
Age (years, mean ± std)	69.38 ± 5.48	72.63 ± 5.35
Height (cm, mean ± std)	156.74 ± 7.50	152.57 ± 7.97
Weight (kg, mean ± std)	61.81 ± 8.38	54.21 ± 6.36
BMI (kg/m^2^, mean ± std)	25.11 ± 2.36	23.33 ± 2.59

**Table 2:** Statistical analysis results of the extracted features including the dynamic plantar pressure features collected throughout the testing process.

Features	Non-Sarcopenia	Sarcopenia	*p*-Value
Age (years)	69.38 ± 5.48	72.63 ± 5.35	0.044 *
Height (cm)	156.74 ± 7.50	152.57 ± 7.97	0.073
Weight (kg)	60.25 (12.00)	52.90 (7.50)	<0.001 ***
BMI (kg/m^2^)	25.11 ± 2.36	23.33 ± 2.59	0.020 *
Pmax1 (kPa)	210.63 (33.71)	252.34 (79.04)	0.016 *
Pmax2 (kPa)	237.16 (28.68)	260.93 (57.21)	0.025 *
Pmax3 (kPa)	279.59 (29.96)	262.13 (13.34)	<0.001 ***
Pmax4 (kPa)	254.14 (21.07)	246.00 (16.02)	0.067
Pmax5 (kPa)	225.77 (46.64)	243.88 (86.98)	0.094
Pmean1 (kPa)	122.64 (27.79)	142.54 (47.05)	0.003 **
Pmean2 (kPa)	145.21 ± 22.39	165.32 ± 25.11	0.007 **
Pmean3 (kPa)	166.82 ± 22.75	169.38 ± 23.96	0.711
Pmean4 (kPa)	156.86 ± 22.09	160.30 ± 21.05	0.582
Pmean5 (kPa)	130.63 (28.75)	160.30 (21.05)	0.004 **
Pstd1 (kPa)	75.80 (15.63)	90.05 (29.21)	0.043 *
Pstd2 (kPa)	88.59 ± 9.89	94.96 ± 15.82	0.082
Pstd3 (kPa)	101.98 (12.23)	94.89 (9.18)	<0.001 ***
Pstd4 (kPa)	91.29 (13.66)	87.98 (11.38)	0.045 *
Pstd5 (kPa)	84.21 ± 12.20	92.11 ± 21.00	0.095
CPmax1 (kPa/kg)	3.59 ± 0.71	4.61 ± 0.88	<0.001 ***
CPmax2 (kPa/kg)	4.04 ± 0.66	4.95 ± 0.73	<0.001 ***
CPmax3 (kPa/kg)	4.73 ± 0.80	5.02 ± 0.80	0.206
CPmax4 (kPa/kg)	4.36 ± 0.80	4.73 ± 0.71	0.089
CPmax5 (kPa/kg)	3.77 (0.84)	4.82 (1.66)	<0.001 ***
CPmean1 (kPa/kg)	2.07 ± 0.45	2.80 ± 0.59	<0.001 ***
CPmean2 (kPa/kg)	2.38 ± 0.41	3.07 ± 0.46	<0.001 ***
CPmean3 (kPa/kg)	2.73 ± 0.45	3.16 ± 0.59	0.009 **
CPmean4 (kPa/kg)	2.57 ± 0.50	3.00 ± 0.50	0.006 **
CPmean5 (kPa/kg)	2.14 (0.61)	2.96 (1.32)	<0.001 ***
CPstd1 (kPa/kg)	1.32 ± 0.27	1.68 ± 0.34	<0.001 ***
CPstd2 (kPa/kg)	1.46 ± 0.27	1.77 ± 0.32	0.001 **
CPstd3 (kPa/kg)	1.73 ± 0.29	1.77 ± 0.30	0.665
CPstd4 (kPa/kg)	1.57 ± 0.32	1.68 ± 0.29	0.260
CPstd5 (kPa/kg)	1.39 ± 0.27	1.71 ± 0.41	0.001 **
contact time (s)	0.35 (0.02)	0.5 (0.10)	<0.001 ***

* *p* < 0.05; ** *p* < 0.01; *** *p* < 0.001.

**Table 3:** Comparison among the proposed prototype system with some existing plantar pressure measurement devices and commercial devices (Co Dev) commonly used for sarcopenia screening.

	Comparison	Preparation Methods	Sensitivity	Response Time	Cost	Portability	Complexity	Wireless Transmission	Real-Time Monitoring
Ref./Co Dev									
[16]		/ ^1^	n.a. ^2^	n.a.	n.a.	√ ^3^	low		√
[20]		/	n.a.	n.a.	n.a.	√	medium	√	√
[21]		/	n.a.	n.a.	high	√	medium		√
[22]		thermal evaporating	n.a.	n.a.	n.a.	√	low		√
[23]		3D-printing	1.19 MPa^−1^	142 ms	low	√	low	√	√
[30]		electrospinning	2.51 mV/(μm ·N)	n.a.	n.a.	n.a.	n.a.		n.a.
[42]		/	n.a.	/	n.a.	√	low		√
DXA		/	/	/	high		high		
BIA		/	/	/	high		high		
CT		/	/	/	high		high		
This work		electrospinning and flexible printing	4.844 mV/kPa	35 ms	low	√	low	√	√

^1^ not mentioned; ^2^ not applicable; ^3^ the device has the corresponding advantage.

## Data Availability

The data presented in this study are available on request from the corresponding author. The data are not publicly available due to restrictions (they contain information that could compromise the privacy of research participants).

## Supplementary Materials

The following supporting information can be downloaded at https://www.mdpi.com/article/10.3390/s24165189/s1, Figure S1: (a) The actual photography of the entire prototype system. (b) The photo of the experimental test bench; Figure S2: (a) The response and recovery time of the sensor. (b) The minimum detection limit of the sensor; Figure S3: The output characteristics of the sensor at 4 bending angles of 15°, 30°, 45°, and 60°; Table S1: The required components and approximate cost for creating a prototype system for measuring plantar pressure established in this article; Video S1: The mobile monitoring interface and the process of measuring plantar pressure during walking.
