# Supplementary material for: Using Flexible-Printed Piezoelectric Sensor Arrays to Measure Plantar Pressure during Walking for Sarcopenia Screening
Source: Sensors (Basel). 2024 Aug 11;24(16):5189. doi: 10.3390/s24165189 (PMC11360066; doi:10.3390/s24165189)
Supplement: Supplementary file 1 [file sensors-24-05189-s001.zip › Supplementary Materials.pdf]

Supplementary Materials

Using Flexible-Printed Piezoelectric Sensor Arrays to Measure Plantar Pressure during Walking for Sarcopenia Screening

Shulang Han [1], Qing Xiao [2], Ying Liang [3], Yu Chen [3], Fei Yan [4], Hui Chen [5], Jirong Yue [6,*], Xiaobao Tian [3,*] and Yan Xiong [1,*]

1 College of Mechanical Engineering, Sichuan University, Chengdu 610065, China; hsl@stu.scu.edu.cn
2 College of Mechanical and Electrical Engineering, Chengdu University of Technology,
Chengdu 610059, China; m_xiao@stu.cdut.edu.cn
3 College of Architecture and Environment, Sichuan University, Chengdu 610065, China;
liangying@scu.edu.cn (Y.L.); yu_chen@scu.edu.cn (Y.C.)
4 Chongqing Municipality Clinical Research Center for Geriatric Diseases, Chongqing University
Three Gorges Hospital, School of Medicine, Chongqing University, Chongqing 404000, China; fei.yan@cqu.edu.cn
5 Department of Senile Medical, The Affiliated Traditional Chinese Medicine Hospital of
Southwest Medical University, Luzhou 646000, China; huige@swmu.edu.cn
6 Department of Geriatrics, West China Hospital, Sichuan University, Chengdu 610041, China
* Correspondence: yuejirong@wchscu.cn (J.Y.); xbtian@scu.edu.cn (X.T.); xy@scu.edu.cn (Y.X.)

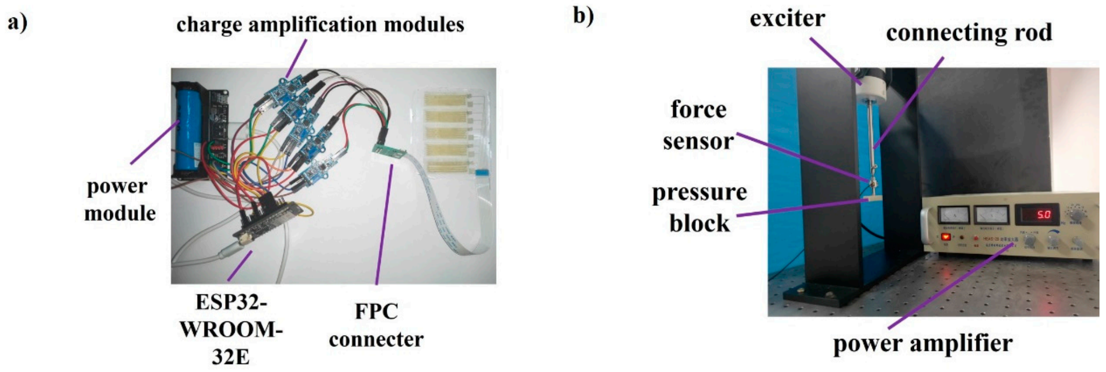

Figure S1. (**a**) The actual photography of the entire prototype system. (**b**) The photo of the experimental test bench.

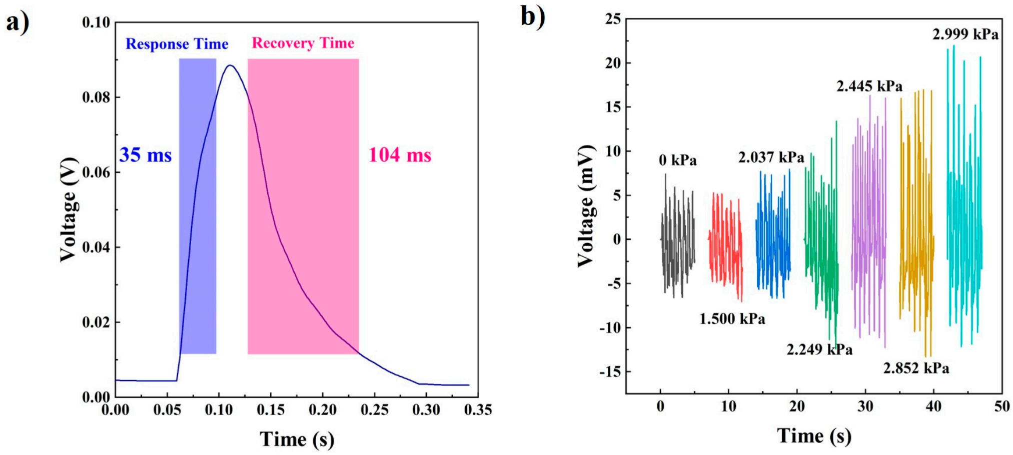

Figure S2. (**a**) The response and recovery time of the sensor. (**b**) The minimum detection limit of the sensor.

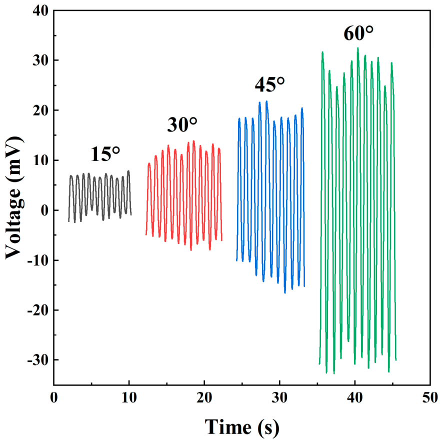

Figure S3. The output characteristics of the sensor at 4 bending angles of 15 °, 30 °, 45 °, and 60 °.

Table S1. The required components and approximate cost for creating a prototype system for measuring plantar pressure established in this article.

Components	Required quantity	Cost
Silicone gel	about 4 ml	about $ 4.13
Silver paste	about 2 ml	about $ 8.94
PET	25 ◊ 25 cm^2	about $ 0.14
FPC line and connecter	1	about $ 0.18
charge amplification module	5	about $ 13.76
ESP32-WROOM-32E	1	about $ 2.08
power module	1	about $ 6.60
**Total**	**about 8 electronic components**	**about $35.83**
